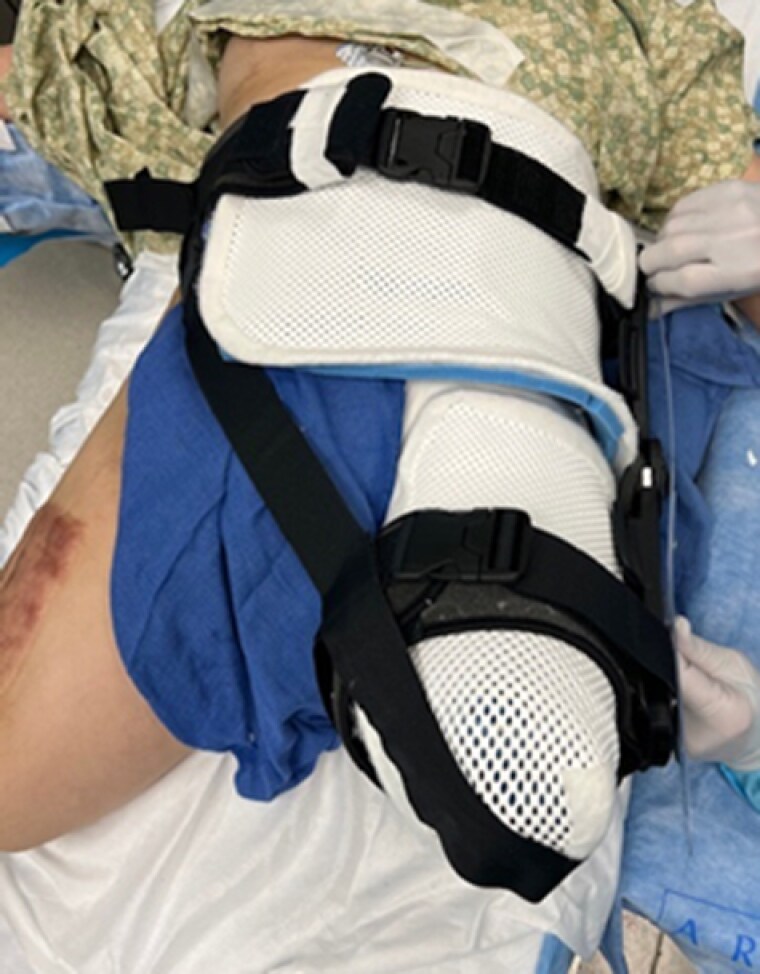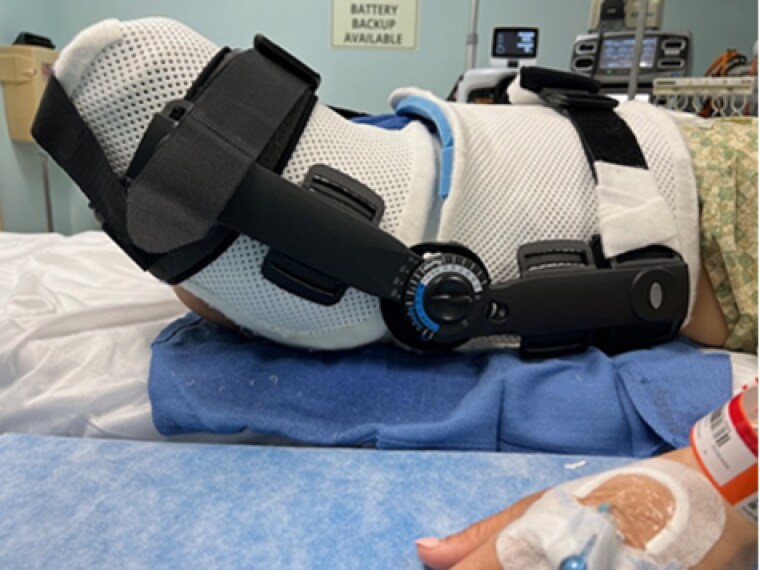# 701 Utilizing a Custom Hinged Orthotic for Management of Hip Flexion Contracture

**DOI:** 10.1093/jbcr/iraf019.330

**Published:** 2025-04-01

**Authors:** Kelsey Windisch, Renee Warthman, Andria Martinez, Derek Murray, Karen Richey, Kevin Foster, Arpana Jain

**Affiliations:** Diane & Bruce Halle Arizona Burn Center; Diane & Bruce Halle Arizona Burn Center; Diane & Bruce Halle Arizona Burn Center; Diane & Bruce Halle Arizona Burn Center; Diane & Bruce Halle Arizona Burn Center; Diane & Bruce Halle Arizona Burn Center; Diane & Bruce Halle Arizona Burn Center

## Abstract

**Introduction:**

The incidence of hip flexion contracture following transtibial and transfemoral amputations is estimated to be 13% and 23%, respectively. The presence of burns and burn scars in the cutaneous field of skin associated with hip flexion complicates contracture prevention. Standard approaches for prevention or management of these contractures include positioning, ROM, exercise, intensive patient education, and in extreme cases, custom orthoses. This is a single case report of a patient with a hip flexion contracture, managed with a novel custom hinged hip extension orthosis following scar resurfacing.

**Methods:**

A 54-year-old female was struck by a semi-trailer at a crosswalk, resulting in bilateral above the knee amputations. She received a split thickness skin graft (STSG) to her L hip during acute reconstruction at an outside hospital. She presented to our institution 3 months later with a hip flexion contracture, severely limiting her mobility, and ability to fit for BLE prosthetics. Under anesthesia, goniometric measurements were taken prior to contracture release and then immediately after resurfacing A custom thermoplastic hinged hip extension orthosis was fabricated utilizing the hinge from an off-the shelf locking, hinged knee brace. The hinge was locked to prevent further hip flexion however was set to allow for increased extension. An additional strap was added to decrease hip abduction. The orthosis was worn at all times for five days. The thermoplastic components of the orthosis were re-fabricated prior to discharge. Goniometric measurements were taken at every healing milestone and weekly in the clinic.

**Results:**

The patient tolerated the orthotic at all times and transitioned to nighttime wear upon discharge. Hip extension progressed from 60 degrees pre-operatively, to 40 degrees immediately following resurfacing to 4 degrees from neutral within one month. The patient is now in the process of being fitted for prosthetics.

**Conclusions:**

Utilizing a custom fabricated hinged hip extension orthosis with thermoplast components allowed for custom adjustments and progressive stretch throughout the course of care. This orthotic is a valuable alternative to the off-the-shelf devices. This custom modification allowed for immediate application following surgical correction of scars, and resulted in compelling gains in range of motion

**Applicability of Research to Practice:**

A custom fabricated hinged hip extension orthosis can progress patient ROM for functional use of prosthetics.

**Funding for the Study:**

N/A